# Paraffin in Surgery

**Published:** 1903-03

**Authors:** 


### PARAFFIN IN SURGERY.

THE distinction of introducing paraffin into surgery is due to Gersung, of Vienna, who some five or six years ago first published his experience with it. Since then it has been employed by many surgeons elsewhere and for a variety of purposes. It has been used to take the place of testes in the scrotum where they have been lost by operation or disease; to fortify relaxed sphincters; to fill out disfiguring or cicatricial depressions. But its principal and most spectacular use has been in the correction of the deformity produced by sunken or "saddle" nose. The cutting or plastic operations for this deformity have proved so far from satisfactory that paraffin injection seems likely to replace them wherever feasible. The quality of paraffin as to consistency is the subject of some dispute, some contending that hard paraffin is to be preferred, as it is under better control in moulding and is less liable to cause thrombosis; while others prefer soft paraffin, that is with a melting point about 110° F., as being more easily injected and less liable to cause sloughing. The operation is said to be devoid of danger if properly performed, though one case of thrombosis (Pfannelstiel) and one of sudden blindness have been reported as being due to it. In the first instance the quantity used was excessive and was very rapidly injected, and in the second the injection was inadvertently made in a vein. The essentials for success in the operation are perfect asepsis, not too much paraffin and adaptability of the tissue.

After injection the paraffin rapidly hardens and becomes like cartilage to the feel. What finally becomes of the paraffin is a matter of conjecture. According to Stephen Paget it is very slowly replaced by fibrous tissue.

Unless further use develops some very serious objection to the method, it promises to be a surgical resource of great utility.

---

INTELLIGENCE is received of the death, in Birmingham by accident, of Dr. W. E. B. Davis. In endeavoring to control an unruly horse at a railroad crossing he was thrown under a passing train and instantly crushed to death. By this tragic incident the South loses one of her most progressive, esteemed and honored sons. Dr. Davis's contributions to surgery are sufficient to give him a name in the annals of medicine. He held many offices of honor in the gift of his fellow-workers and graced them all. His sudden and deplorable death will create a breech in the ranks difficult to fill.

---

DR JOHN GERDINE died at Athens, Ga., of pneumonia, February 19th. Dr. Gerdine was one of the best known and most highly honored physicians in his section of the country, highly esteemed by profession and laity alike. He was an old and valued patron of this journal, and we much regret to learn of his death.

